# Shifting Baselines, Local Impacts, and Global Change on Coral Reefs

**DOI:** 10.1371/journal.pbio.0060054

**Published:** 2008-02-26

**Authors:** Nancy Knowlton, Jeremy B. C Jackson

## Abstract

The striking health of remote coral reefs provides clear evidence that protection from local overfishing and pollution can help mitigate the impacts of global warming.

Imagine trying to understand the ecology of tropical rainforests by studying environmental changes and interactions among the surviving plants and animals on a vast cattle ranch in the center of a deforested Amazon, without any basic data on how the forest worked before it was cleared and burned. The soil would be baked dry or eroded away and the amount of rainfall would be greatly decreased. Most of the fantastic biodiversity would be gone. The trees would be replaced by grasses or soybeans, the major grazers would be leaf-cutter ants and cattle, and the major predators would be insects, rodents, and hawks. Ecologists could do experiments on the importance of cattle for the maintenance of plant species diversity, but the results would be meaningless for understanding the rainforest that used to be or how to restore it in the future.

Fortunately, ecologists began to carefully describe tropical forests more than a century ago, and vast areas of largely intact forests have persisted until today, so there are meaningful baselines for comparison. Networks of 50-hectare plots are monitored around the world [1], and decades of experiments have helped to elucidate ecological mechanisms in these relatively pristine forests [2]. But the situation is very different for the oceans, because degradation of entire ecosystems has been more pervasive than on land [3] and underwater observations began much more recently. Monitoring of benthic ecosystems is commonly limited to small intertidal quadrats, and there is nothing like the high-resolution global monitoring network for tropical forests for any ocean ecosystem.

This lack of a baseline for pristine marine ecosystems is particularly acute for coral reefs, the so-called rainforests of the sea, which are the most diverse marine ecosystems and among the most threatened [4–8]. Most of the world's tropical coastal oceans are so heavily degraded locally that “pristine” reefs are essentially gone, even if one ignores changes associated with already rising temperatures and acidity [3]. Most modern (post-SCUBA) ecological studies have focused on reef ecosystems that are moderately to severely degraded, and we have a much better understanding of transitions between human-dominated and collapsed reefs than between human-dominated and quasi-pristine reefs. Even the classic studies of Caribbean reefs that began in the 1950s were based on reefs that had very high coral cover but were severely overfished, and the first systematic surveys of subtidal Australian reefs in the late 1960s began after a severe outbreak of the crown-of-thorns starfish Acanthaster planci had devastated coral populations along much of the Great Barrier Reef. We are thus left without a clear understanding of how reefs functioned in the absence of major human impacts.

This is the problem of shifting baselines [3,9], which is at the root of ongoing controversy about the relative importance of and synergies among the major factors driving coral reef decline (overfishing, land-based pollution, and global change) and what, if anything, can be done to stop it. Coral reefs are physically dynamic constructions, with living corals and other calcifying organisms secreting new skeletons and older skeletons eroding into sand. Thus reefs can only persist as substantial physical structures if net growth remains positive [10], and factors that decrease growth and reproduction or increase mortality of corals have the potential to tip the balance toward inexorable reef decline (Figure 1A and 1B). This is what we have seen over the past few decades, as living coral cover has decreased on average by one-third to more than two-thirds worldwide [11,12].

**Figure 1:**
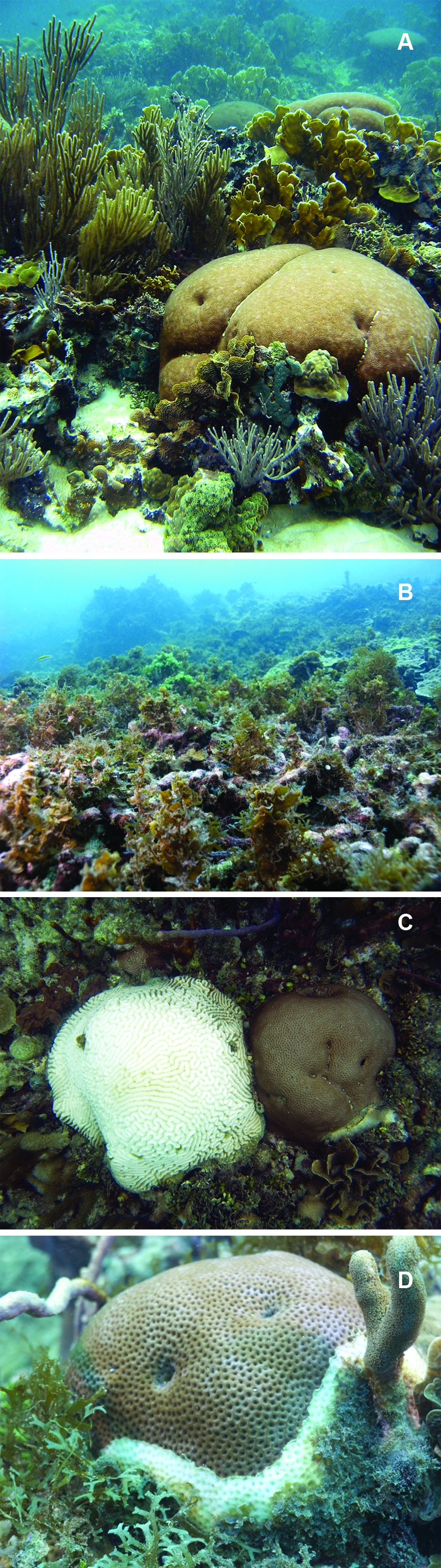
Healthy Reefs, Dying Reefs, and Corals in Bocas del Toro, Panama (A) Example of a healthy reef with abundant living coral. (B) Example of a reef in which most coral has died and been replaced by macroalgae. (C) Bleached and healthy coral colonies; both are alive but the bleached colony has lost its symbiotic algae. (D) Coral suffering from disease and with encroaching macroalgae. See Sandin and colleagues [39] for analogous images from the Northern Line Islands. (Photo credit: David Kline, Centre for Marine Studies, University of Queensland, Australia).

## Why Reefs Decline—Local versus Global Impacts

The greatest scientific uncertainties in the debate about coral reef decline concern interactions between local versus global disturbance, a debate that is aggravated by the tendency among reef ecologists to attribute changes to single factors [13] rather than the synergies among them [7,14]. There is, however, every reason to believe that the extent of local impacts may affect the responses of corals and other reef organisms to global change [7]. Global changes, most importantly warming and acidification, have already occurred and will continue, even under the most optimistic of scenarios, so that conservation strategies must be evaluated accordingly [5–7,15]. Warming causes coral bleaching (Figure 1C), which is the breakdown in the symbiosis between corals and their symbiotic dinoflagellates (zooxanthellae), which are essential for coral growth. Acidification decreases calcification and may ultimately result in the inability of corals to form a skeleton. Local and regional impacts predate warming and acidification by decades to millennia. Of particular importance are the effects of resource extraction and lowered water quality on reef ecosystems and their effects on corals via overgrowth of macroalgae and disease (Figure 1D) [6,7,16–18].

Trophic structure, biodiversity, resistance, and resilience are key attributes of all reef ecosystems. Understanding how they respond to increasing local human impacts and recover with decreasing local human impacts is thus essential for planning conservation strategies [4,6,19]. Yet at the moment, we have little understanding of even the basic shape of these relationships across a truly broad spectrum of human influence. This is a difficult problem because of the following: (1) the large number of interactions among species, many likely to be nonlinear [15,19–21], (2) the many noncongruent spatial and temporal scales associated with degradation and recovery for different members of the community [19,22], and (3) the likelihood that degradation and recovery will follow different trajectories [7,19,20].

Paleontological studies provide important insights about what pristine reefs were like for groups like corals with a good fossil record [23,24], and archeological and historical analyses are particularly useful for conspicuous or economically important taxa [3]. Nevertheless, many ecologists are skeptical of historical data in the absence of experiments and question the importance of the shifting baselines syndrome [9] for understanding how pristine ecosystems functioned before human disturbance [3]. However, small-scale experiments are of limited utility for evaluating conservation options, and large-scale experiments are impractical or unethical at ecologically appropriate scales. The important exception, to which we will return at the end, is that large-scale management decisions are in themselves ecological experiments that can provide important insights, albeit without the usual replication and controls.

## Comparing Reefs with and without People

Two complementary strategies to compare reef ecosystems across strong gradients of recent human disturbance can help to resolve this dilemma if adopted on suitably large spatial and temporal scales. Both exploit the extremes of reef condition to disentangle cause and effect, rather than the average condition characterized by meta-analyses [11,12]. The first and most popular approach uses comparisons of sites inside and outside of marine reserves. Such comparisons can be treated as experiments, although there are problems due to wide variation in the size, age of protection, and actual extent of protection of reserves. In addition, most reserves are smaller than the home ranges of major consumers and have existed for much less time than the generation times of ecologically important corals [22,25]. Even large, old reserves are embedded in regions of overfishing and therefore lack pristine abundances of apex predators [25].

The second approach compares sites across broad gradients of human population size at numerous locations, taking advantage whenever possible of the few extant reefs that approach pristine conditions because of their remote location and hence low economic value. These reefs represent a virtual “time machine” for the descriptive and experimental comparison of ecological processes and resilience on minimally disturbed reefs, and provide a more ecologically meaningful baseline for comparison of reef ecosystems across gradients of human disturbance [26, 27]. This approach has the advantage of comparisons on ecologically more realistic spatial scales, but has the disadvantage that gradients in human population are inevitably imbedded in other physical or oceanographic gradients, so that local impacts are confounded with regional environmental patterns.

Comparisons between reefs inside and outside of reserves can provide insights to processes of recovery, because reserves are typically established in response to degradation that has already occurred. In contrast, comparisons among unpopulated and densely populated regions are probably the only way to observe in real time the actual processes of degradation from once-pristine conditions. Together, these approaches help to illuminate how marine ecosystems behave across a full spectrum of human impacts at scales relevant for conservation. Recent studies illuminate trajectories of both initial loss following human disturbances and recovery following protection across a wide range of levels of degradation (Table 1) and help to identify observations and experiments needed to test alternative hypotheses. Although uncertainties remain, these studies show that local conditions must be taken into account when evaluating the impacts of global change.

**Table 1:**
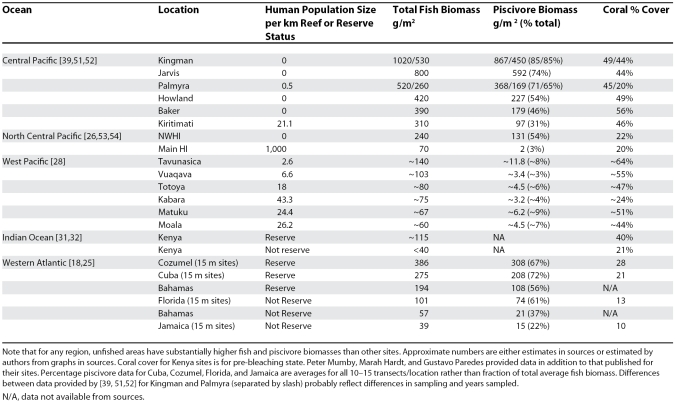
Summary Statistics for Some of the Sites in the Studies Reviewed, Ranked in Order of Total Fish Biomass

### Trophic structure.

Fishes are the best-studied taxon, and by and large, variations in fish biomass and trophic structure are qualitatively consistent with common sense: more people (or more people fishing) result in fewer fish. Apex predators are typically affected most because of their life histories, because they are targeted first, or because most reserves are too small to protect them effectively. Fish biomass on remote, uninhabited, and protected atolls in the Central Pacific Ocean and Northwestern Hawaiian Islands is several times to more than an order of magnitude greater than that on well-studied reefs in the Western Pacific Ocean, Indian Ocean, and Caribbean Sea, most of which are unprotected and have been severely overfished (Table 1). Among Pacific islands, these patterns are clearly related to the numbers of people on each island, regardless of widely varying oceanographic conditions.

High fish biomass is typically associated with low cover and biomass of macroalgae (although biomass is rarely measured), but coral and coralline algal abundances are more variable and not tightly correlated with high fish abundance [18,25,27–33]. Nevertheless, low abundance of corals and coralline algae is almost invariably associated with high abundance of fleshy or turf macroalgae. The causes are complex because so many interacting factors, including overfishing, pollution, and warming, can kill corals directly as well as promote growth of macroalgae that can also kill corals directly by overgrowth or indirectly by promoting coral disease [34–36].

Indirect ecological effects, including trophic cascades, are a common and seemingly logical explanation for the co-occurring patterns in the fish and benthic communities associated with fishing [37]. Theoretically and empirically, it is clear that removal of herbivores can often increase algal cover at the expense of corals [17,18,25,27–33]. However, removal of apex predators might be expected to have the opposite effect if the consequence were the reduction in their herbivorous prey (e.g., [18]). However, this prediction is qualified by such factors as the relationship between herbivore size and grazing impacts, the spatial and temporal scale over which fishing is reduced, and the complexity of reef fish food webs. Although modeling studies suggest that the removal of top predators may cause trophic cascades in linear food chains with sharks at the top and herbivorous fish at the bottom [38], there is little empirical evidence that large numbers of apex predators result in decreased biomass of herbivores or decreased herbivory, even in the presence of large numbers of apex predators [18,25,29,39]. In fact, the best evidence for trophic cascades suggests that protection of reef fishes leads to a reduction in the number of sea urchins and an increase in calcifying algae [31], and a reduction in the number of crown-of-thorns starfish and an increase in corals [27]—both of which are positive developments for reef construction.

### Biodiversity.

Loss of biodiversity is potentially permanent if it is the result of global extinction, and may also affect ecosystem services, resistance, and resilience [40]. Ironically, despite long-standing interest in and concern about biodiversity loss, we know very little about how human impacts affect species diversity on coral reefs [6]. In part, this stems from the enormous number of species associated with coral reefs, many of which cannot be reliably identified due to lack of taxonomic expertise or because they remain undescribed [41]. As a consequence, either a handful of taxonomic groups (especially corals and fishes) currently stand as proxies for species biodiversity broadly [42], or biodiversity is evaluated at coarser taxonomic levels [28].

The Intermediate Disturbance Hypothesis [43] predicts that diversity should increase at low levels of human disturbance as competitively dominant species are suppressed, and then decrease as disturbance increases to severe levels that are harmful to larger numbers of species. However, the only published studies of differences in biodiversity across gradients of human disturbance are for already affected reefs; under these conditions, we would expect consistent decreases in diversity with increasing disturbance because there is no undisturbed (“pristine”) baseline for comparison. In line with this prediction, Dulvy et al. [28] found that the diversity of mobile epifauna declined, both in terms of richness and evenness, with increasing human impacts, and McClanahan et al. [31,32] reported a 2-fold difference in fish diversity, and a somewhat smaller difference in coral generic richness, between protected and unprotected areas.

However, new surveys from the Northern Line Islands [39] demonstrate that the patterns are more complex than expected from Connell's hypothesis. Species richness for fishes increased with increased human populations and disturbance, and peaked on the atoll with the largest human population, whereas species richness for corals consistently decreased across the same gradient. Interpretation of these diversity patterns is complicated by the different atoll sizes and the substantial differences in life history characteristics of fishes and corals. But taken at face value, they suggest that coral diversity might be more sensitive than fish diversity, responding to even relatively light levels of human impacts.

### Resistance and resilience.

Resistance and resilience are measures of the ability of ecosystems to withstand or recover from anthropogenic and natural stresses. For coral reefs, the most important data concern the corals themselves, since they provide the three-dimensional structure upon which much of the entire reef ecosystem depends, either directly or indirectly [6,10,36]. Of particular interest is whether the changes associated with local human impacts affect the ability of corals to withstand the negative effects of physical factors (increasing storms, temperature, and acidity) or biological factors (changes in competitors, predators, pathogens, and invasive species), or to recuperate from mortality events via recruitment.

The best-understood aspects of coral resistance and resilience relate to the effects of overfishing, degraded water quality, and increased macroalgal abundance on coral recruitment (resilience) and coral disease (resistance). Many corals require hard substrates (and in particular, coralline algae) to recruit, and the relationship between recruitment failure and increasing macroalgal dominance due to loss of herbivory, and the converse, are well documented [35,44]. Large amounts of macroalgae may also destabilize microbial communities [45], either by changing water chemistry near coral surfaces [46] or by serving as a reservoir for pathogens [47]. High anthropogenically derived nutrient levels could also simultaneously increase macroalgae and disease [34].

The recent survey of the Northern Line Islands documents decreased coral recruitment, increased coral disease, and increased abundance of microbes (including potential pathogens) with increasing human population size [39,48]. However, there are no data to suggest that corals become less vulnerable to bleaching with reduced local impacts, so that the high cover of living corals on uninhabited Central Pacific atolls may reflect rapid recovery (resilience) rather than resistance. Indeed, in Kenya, differences in coral cover between protected and unprotected areas disappeared shortly after the 1998 major bleaching event [32]. Recent work in the Bahamas [33] also demonstrates that protection from fishing is associated with more coral recruitment and hence potentially greater resilience, although this has not yet translated into markedly increased coral cover. The failure of corals to increase presumably reflects the long generation times and slow growth of corals [22,25], as well as regional events that can set back coral recovery such as coral bleaching and severe hurricanes. Finally, the potential interactions between acidification—the other major global impact affecting reefs—and local human impacts remain largely unexplored [5].

## Implications of Near-Pristine Baselines for Conservation and Conservation Science

New insights in science often come from examining the exceptions to general patterns rather than the norms. The remote, uninhabited atolls of the Central Pacific are a case in point and cause for cautious optimism. Despite increased warming and coral bleaching throughout the Pacific, these reefs still support extraordinarily abundant fish populations dominated by apex predators and among the highest reported abundances of living coral and coralline algae [11,12,39]. This is as true for atolls in less nutrient-rich waters, like Kingman and Palmyra, as for atolls in highly productive upwelling regions, like Jarvis, Howland, and Baker. Detailed studies are lacking to determine whether these reefs have somehow escaped massive bleaching or, as we believe more likely, have more successfully recovered from bleaching due to high recruitment and rapid growth of corals, and lower levels of macroalgal overgrowth, coral disease, and outbreaks of coral predators [27,34,39,45,48]. But regardless of the ultimate explanation, the simple persistence of these luxuriant reefs is fundamentally inconsistent with the growing belief that the effects of global change are so overwhelming that other factors can be largely ignored [13].

**Figure 2:**
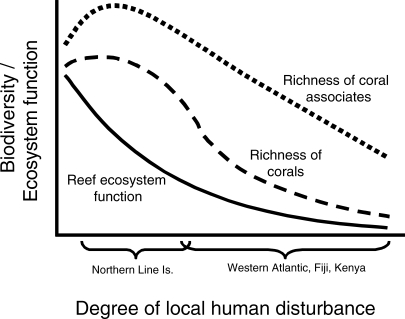
Inferred Relationships between Local Human Disturbance and Various Ecosystem Attributes, as Evidenced by Studies Reviewed

There is, however, no room for complacency. Most reefs are not yet as degraded as cattle ranches in the Amazon, but they are poised at the brink [4–7,11,12]. Very small numbers of people can have a big impact on trophic structure (Table 1) and ecosystem resistance and resilience, which may degrade much faster than biodiversity. Figure 2 illustrates the inferred relationships between the intensity of local anthropogenic disturbance and biodiversity and ecosystem function based on the studies reviewed in this essay. Most surprisingly, given the substantial attention of conservationists to “hot spots” of biodiversity [42], ecosystem function appears to decline long before any substantial decline in biodiversity. This is especially apparent for the diversity of fish species on the Northern Line Islands reefs that is negatively correlated with that of reef corals [39]. Thus, corals may be more sensitive to extinction due to human impacts than their associated species that can move to other habitats, an inference that is consistent with the observation that reef fishes recover rapidly following protection whereas corals may require several decades or more [25,31–33].

Marine protected areas are only effective if they are large, well enforced, and have good water quality. Moreover, no amount of local management can protect against ever increasing global impacts indefinitely. Even stress-resistant zooxanthellae have upper temperature limits [49] and, although corals may migrate to higher latitudes, they may disappear throughout most of their original range. Likewise, corals with weakened skeletons due to ocean acidification will be less resistant to storms and bioerosion, and corals without skeletons may not survive in the wild despite their survival in the laboratory [50] and certainly cannot contribute to reef construction. Thus, reef formation could halt entirely.

In the face of these daunting uncertainties, coral reef ecology needs to be more focused and coordinated on a global scale, with research strategies comparable to the network of tropical forest studies that makes all data available in a consistent and easily accessible format [1]. Existing monitoring networks [8] have played a vital role in alerting scientists and the public to the magnitude of decline, but they are not set up to provide the kinds of rigorously detailed scientific data required to address fundamental questions for the future (Box 1); and the same is true of meta-analytic surveys, no matter how rigorous and detailed, because the data were not collected for the purpose [11,12].

**Box 1. Pressing Questions Regarding the Importance of Local Management to Conserve Coral Reefs in the Context of Global Change**

1. To what extent do overfishing and eutrophication increase the vulnerability of reef corals to bleaching, disease, and acidification caused by global climate change; and, conversely, does protection from these local stressors decrease the vulnerability of reef corals to the effects of climate change?

2. If local protection decreases the vulnerability of corals to climate change, what are the physiological or ecological mechanisms involved, including changes in associated microbial populations and their interactions with their coral hosts?

3. Does protection from overfishing and eutrophication increase rates of coral recruitment, growth, and reproduction that are essential to the reestablishment of coral communities following mass mortality due to the effects of climate change or natural disturbance?

4. Can we identify critical breakpoints and thresholds in the abundance and trophic composition of marine consumers below which coral populations will inevitably decline or fail to recover?

More nearly pristine reefs, such as the uninhabited atolls in Table 1, are of vital importance in the design of such research networks for the future, both as monitoring stations for the impacts of global change under ecologically optimal conditions of minimal local human impact, and as sites for observations of ecological processes and experiments. In addition, networks of very large fully protected areas, such as the newly zoned Great Barrier Reef and the Northwestern Hawaiian Islands [7], are also needed for meaningful assessment of the potential for reef recovery and restoration under different management strategies. Such protection is the special responsibility of developed countries that have the financial and alternative food resources to help to put some areas completely off limits. In contrast, some combination of traditional management, or co-management by traditional societies in partnership with nongovernmental organizations, is probably the best available option in areas of more limited resources [14].

In sum, local actions do make a difference, not only to fishes, but also to reef ecosystems as a whole, and they do so across the entire spectrum of local human impacts and oceanographic conditions where reefs occur. The areas of biggest concern for the immediate future are apex predators at the top, because they are globally so rare, and corals at the bottom, because of their continuing decline, apparent vulnerability to even modest local human impacts, and extreme sensitivity to all aspects of global change. Both risk extinctions if nothing is done to halt their global downward trajectories. Coral reefs are but one of many reasons for reducing and reversing global change, and the threat posed by carbon emissions to the well-being of humans and the planet is enormous and ever-growing. Nevertheless, how to manage coral reefs locally in a globally changing world so that they retain or regain the critical ecosystem attributes of uninhabited reefs and still meet human needs is the central challenge facing reef conservation today.
